# Supplementary material for: Representation of multiple objects in macaque category-selective areas
Source: Nat Commun. 2018 May 2;9:1774. doi: 10.1038/s41467-018-04126-7 (PMC5932008; doi:10.1038/s41467-018-04126-7)
Supplement: Supplementary file 1 — SUPPLEMENTARY INFO [file 41467_2018_4126_MOESM1_ESM.pdf]

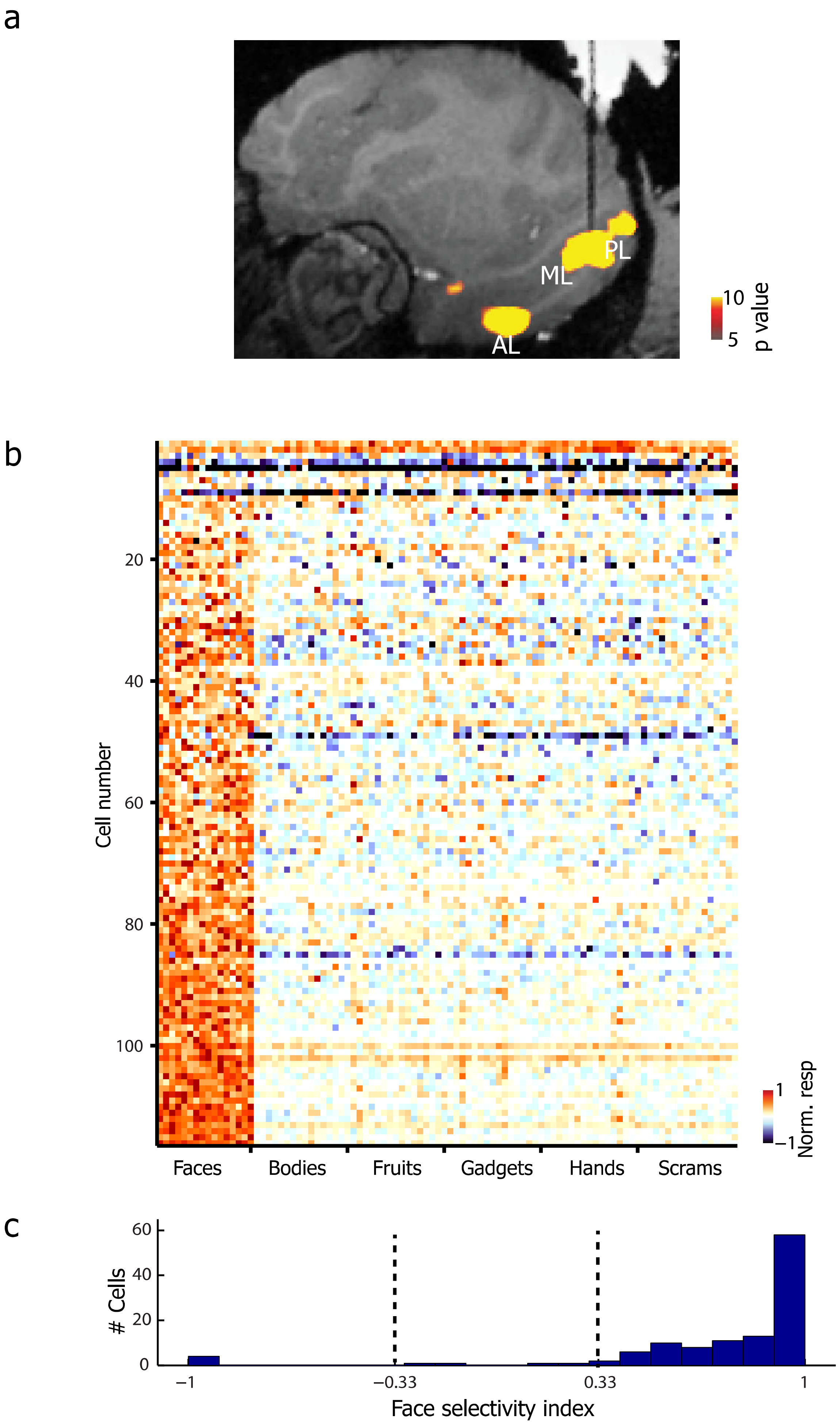
a
PL
ML
AL
10
5
p value
b
Cell number
20
40
60
80
100
Faces
Bodies
Fruits
Gadgets
Hands
Scrams
1
−1
Norm. resp
c
Cells
60
40
20
0
−1
−0.33
0.33
1
Face selectivity index

Supplementary Figure 1. Localization of face patches

(**a**) Sagittal slice showing the location of fMRI-identified face patches in one monkey targeted for recording; dark black line indicates electrode. Stereotactic coordinates for the location of ML were as follows: M1 AP 4.50, ML 25.5 (right hemisphere); M2 AP 4.50, ML 27.43 (right hemisphere), M3 AP 4.00, ML -26.2 (left hemisphere). (**b**) Neuronal responses (baseline-subtracted, averaged from 60 to 220ms) to images of different categories recorded from the ML. (**c**) Distribution of FSI (see Methods) across all visually responsive neurons. Dotted lines indicate |FSI|=0.33.

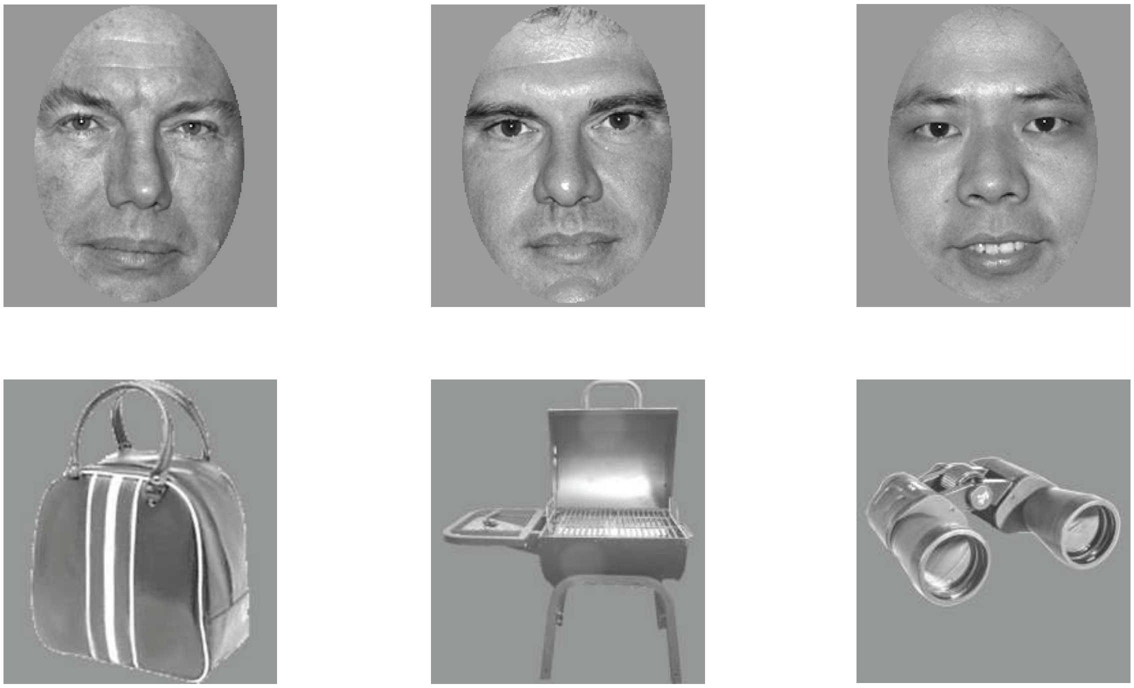

Supplementary Figure 2. Stimuli for face-object pair experiment

The face images are from FERET face data base. The object images shown are similar to the objects actually shown, but due to copyright reasons, we cannot show the original. The grill image is adapted from an image from flickr.com (https://www.flickr.com/photos/fictures/14808488890/).

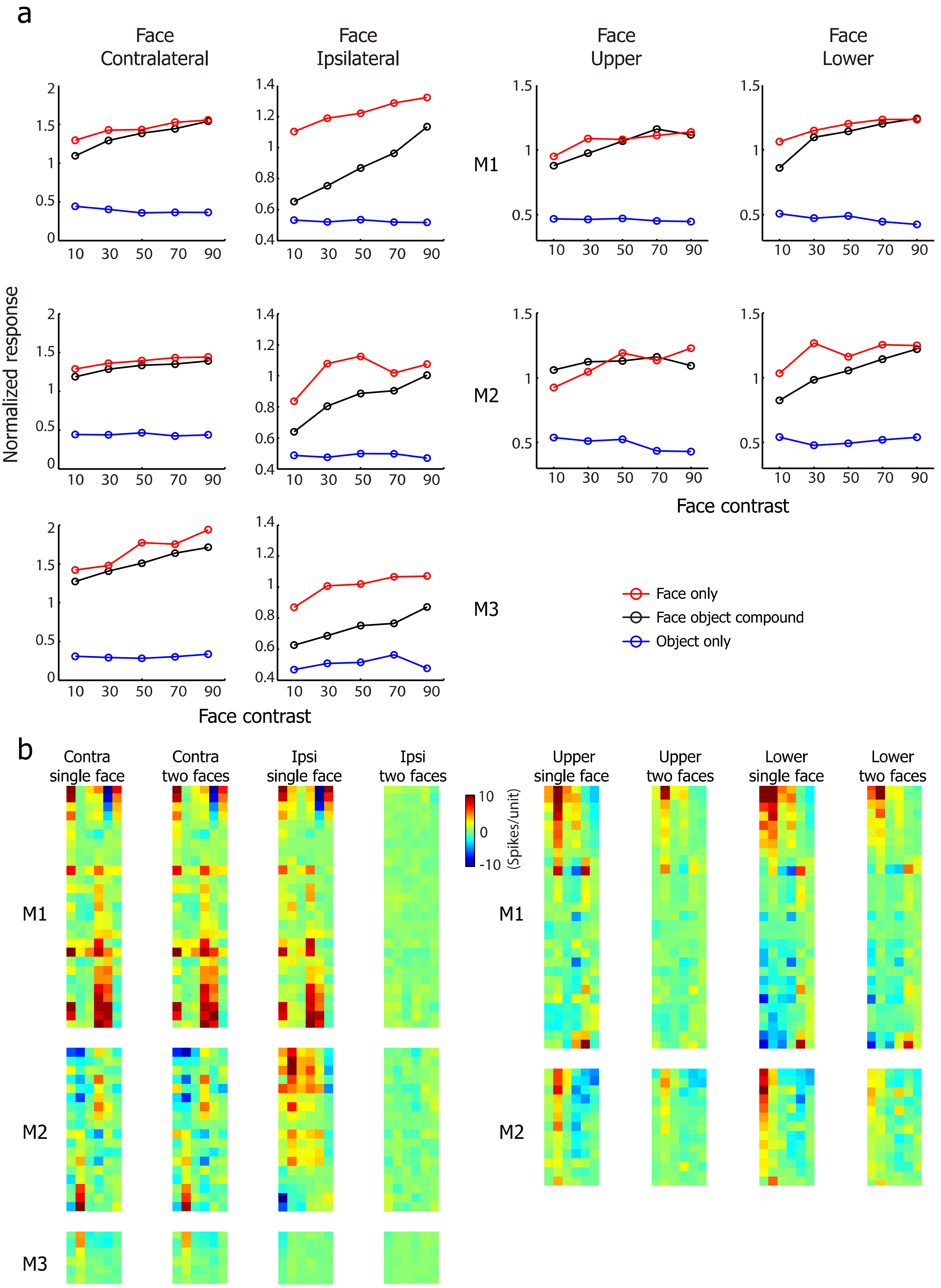

a
Face Contralateral
Face Ipsilateral
Face Upper
Face Lower
Normalized response
M1
M2
M3
Face contrast
Face only
Face object compound
Object only
b
Contra single face
Contra two faces
Ipsi single face
Ipsi two faces
Upper single face
Upper two faces
Lower single face
Lower two faces
10
0
-10
(Spikes/unit)

Supplementary Figure 3. Main results shown separately for three monkeys. (**a**) Results of face-object experiment for monkeys M1, M2, M3. Conventions as in Figure 2a, d, g, j. (**b**) Results of face-face experiment for monkeys M1, M2, M3. Conventions as in Figure 4b, d.

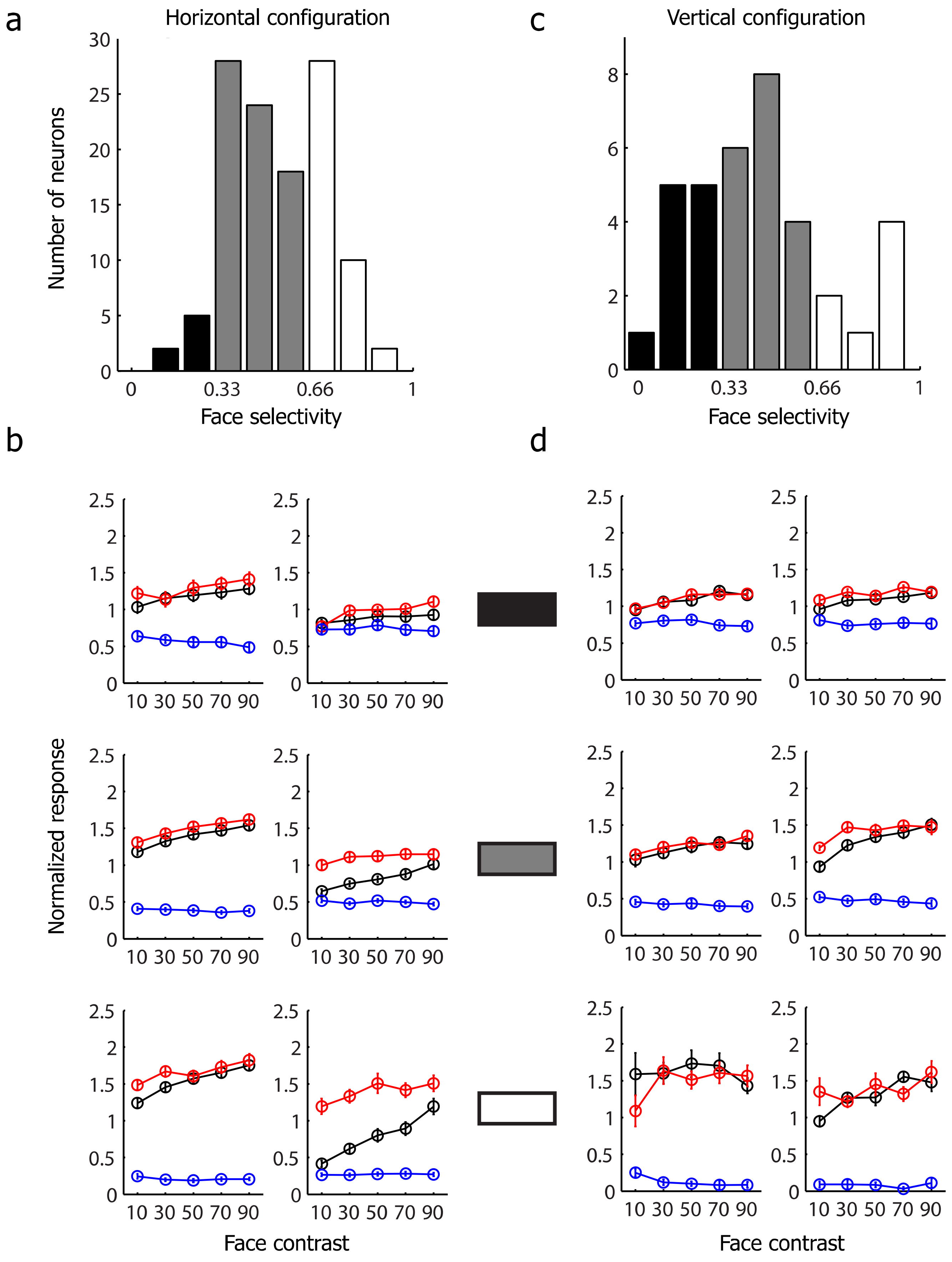
a
Horizontal configuration
Number of neurons
Face selectivity
c
Vertical configuration
Face selectivity
b
d
Normalized response
Face contrast
Face contrast

Supplementary Figure 4. The integration rule for face-object pairs does not depend on a neuron's face selectivity

(**a**) The distribution of face selectivity, defined as FSI, across the population in the horizontal configuration. Neurons were classified into three groups: (1) low face selectivity (FSI < 0.33 black), (2) medium face selectivity (0.33 < FSI <0.66), (3) high face selectivity (FSI > 0.66, white). (**b**) The population mean response of each group defined in (a). Conventions as in Figure 2a, d. (**c**) Same as (a) for the vertical configuration. (**d**) The population mean response of each group defined in (c). Conventions as in Figure 2g, j.

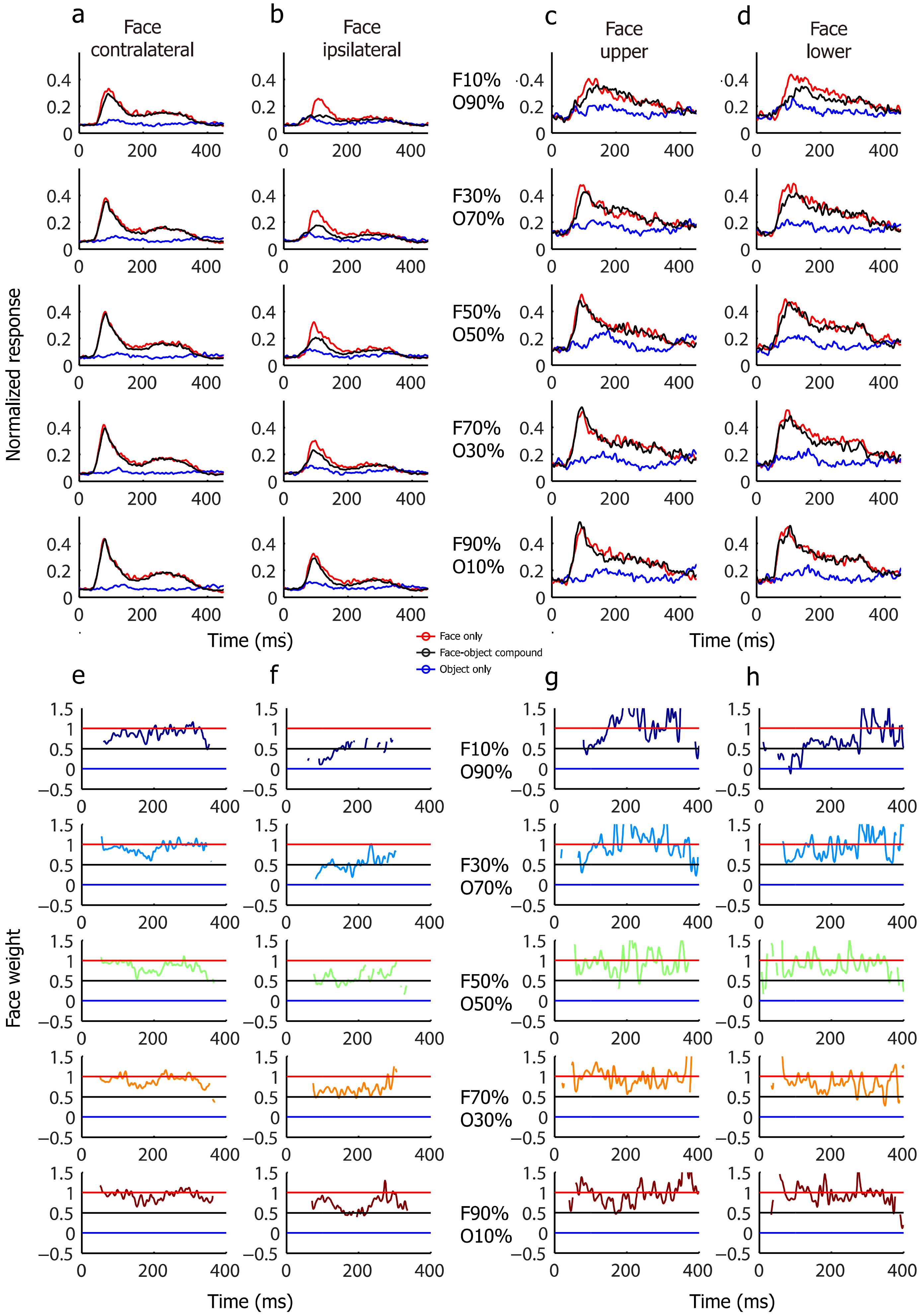
a
Face contralateral
b
Face ipsilateral
c
Face upper
d
Face lower
F10% O90%
F30% O70%
F50% O50%
F70% O30%
F90% O10%
Normalized response
Time (ms)
Face only
Face-object compound
Object only
e
f
g
h
Face weight
Time (ms)

Supplementary Figure 5. Response time course in response to face-object pairs. (a) The population mean response of neurons in ML to face-object pairs with face presented in the contralateral visual field and object presented in the ipsilateral visual field. Black lines represent the responses to the face-object pairs across time. Red (blue) lines represent the responses across time to the constituent face (object) of the corresponding face-object pair when presented in isolation. The 5 rows represent 5 different contrast levels. **(b)** Same as (a), for the condition in which faces were presented in the ipsilateral visual field and objects were presented in the contralateral visual field. **(c)** Same as **(a)**, for the condition in which faces were presented in the upper visual field and objects were presented in the lower visual field. **(d)** Same as (a), for the condition in which faces were presented in the lower visual field and objects were presented in the upper visual field. **(e)** The face weight (see Methods) computed across time at five relative contrast levels (from top to bottom) with face presented in the contralateral visual field and object presented in the ipsilateral visual field. Note some time points are missing because the difference between the responses to face and object was too small (<0.04), making the weight estimation meaningless. **(f)** Same as (e), for the condition in which faces were presented in the ipsilateral visual field and objects were presented in the contralateral visual field. **(g)** Same as (e), for the condition in which faces were presented in the upper visual field and objects were presented in the lower visual field. **(h)** Same as (e), for the condition in which faces were presented in the lower visual field and objects were presented in the upper visual field.

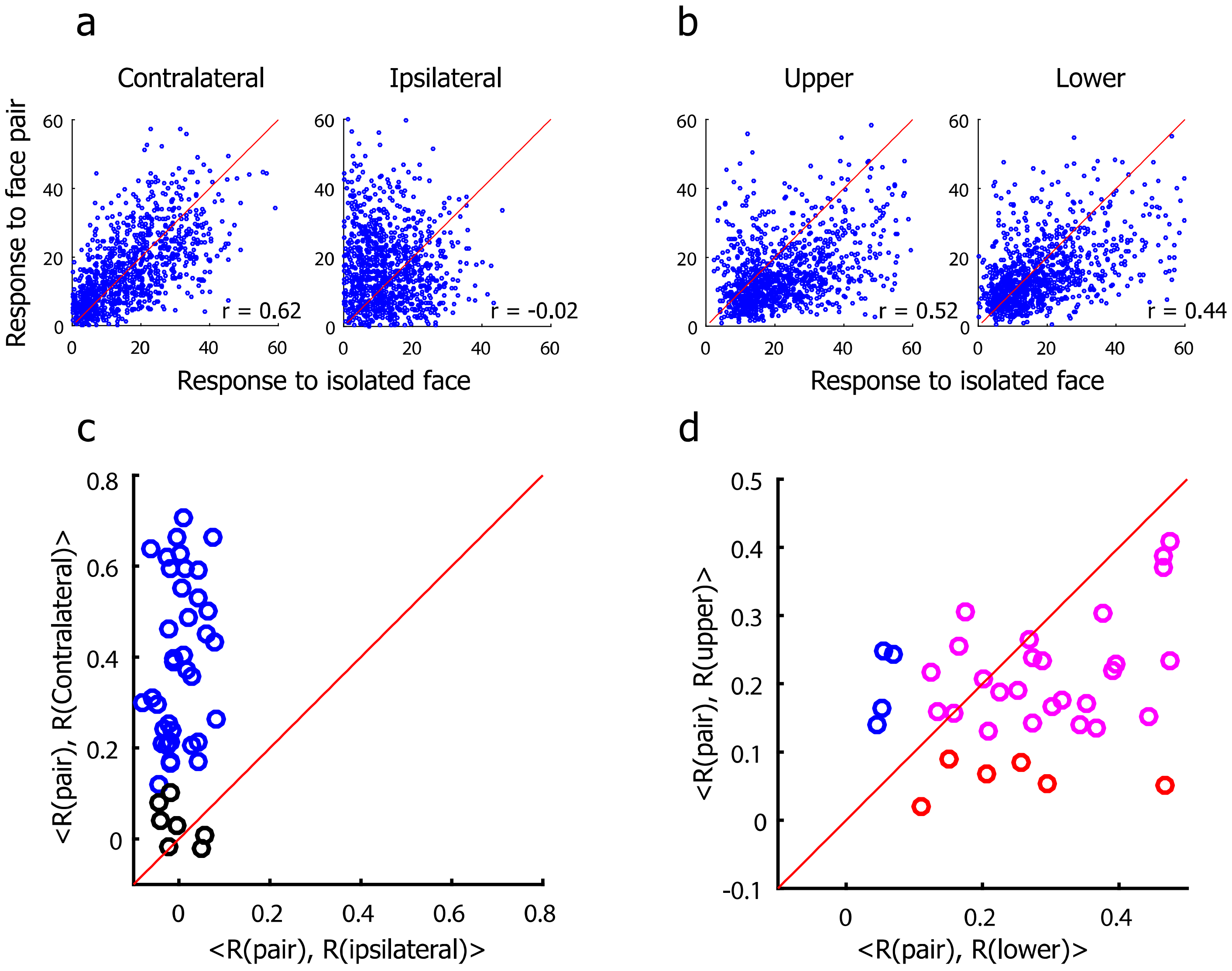
a
b
Contralateral
Ipsilateral
Upper
Lower
Response to face pair
r = 0.62
r = -0.02
r = 0.52
r = 0.44
Response to isolated face
Response to isolated face
c
d
<R(pair), R(Contralateral)>
<R(pair), R(ipsilateral)>
<R(pair), R(upper)>
<R(pair), R(lower)>

Supplementary Figure 6. The correlation between response magnitudes to pairs of faces to isolated faces

(**a**) Response magnitudes to pairs of faces versus to isolated faces (left: contralateral face, right: ipsilateral face) for one example cell, with faces in the horizontal configuration. **(b)** Response magnitudes to pairs of faces versus to isolated faces (left: upper face, right: lower face) for one example cell, with face in the vertical configuration. **(c)** Scatter plots of the correlation between responses to pairs of faces and contralateral isolated faces versus the correlation between responses to pairs of faces and ipsilateral isolated faces, across all neurons. Blue indicates neurons that showed a significant correlation between the responses to pairs of faces and to contralateral isolated faces. Black indicates neurons that showed no significance for either correlation. ($p < 0.05$, Bonferroni corrected). **(d)** Scatter plots of the correlation between responses to pairs of faces and isolated faces above fixation versus the correlation between responses to pairs of faces and isolated faces below fixation across all the neurons. Blue indicates neurons that only showed a significant correlation between the responses to pairs of faces and isolated faces above the fixation. Red indicates neurons that only showed a significant correlation between responses to pairs of faces and isolated faces above fixation. Magenta indicates neurons that showed significance for both correlations ($p < 0.05$, Bonferroni corrected).

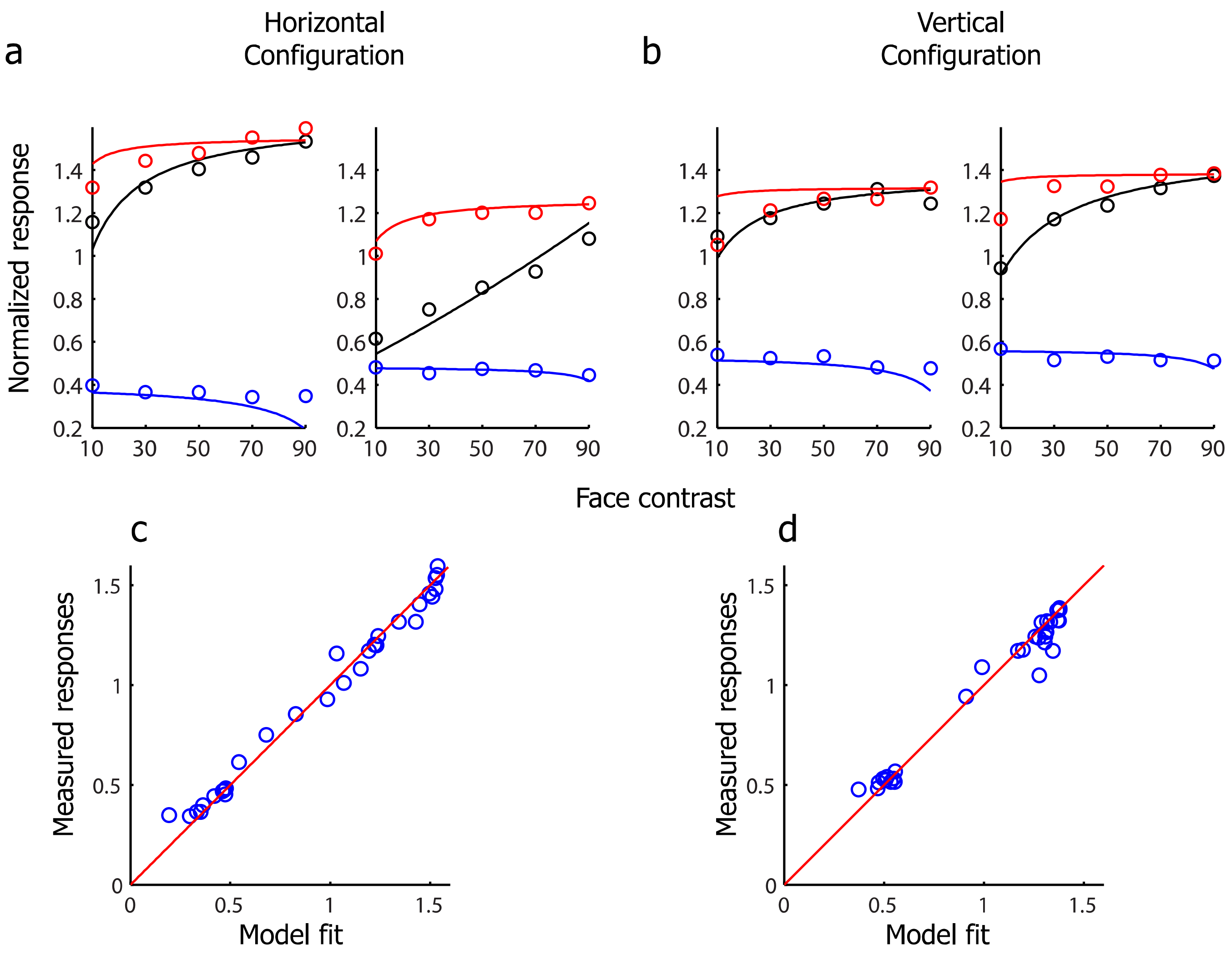
a
Horizontal Configuration
b
Vertical Configuration
Normalized response
1.4
1.2
1
0.8
0.6
0.4
0.2
10
30
50
70
90
Face contrast
c
d
Measured responses
Model fit
1.5
1
0.5
0

Supplementary Figure 7. The normalization model using LFP amplitudes as weights predicts neural responses. (a) The normalization model fit using LFP amplitudes as weights to the face-object experiment when faces and objects were presented horizontally. Line indicates model fit, circles indicate observed data (data same as Figure 2b, e). **(b)** Same as (a), for faces and objects presented vertically (data same as Figure 2h, k). **(c)** The correlation between the observed responses and the normalization model fit when faces and objects were presented horizontally. We added a free LFP baseline parameter to obtain the model fits (i.e., the weight in the normalization model were calculated as the LFP amplitude minus the baseline). **(d)** Same as (c), for face and objects presented vertically.

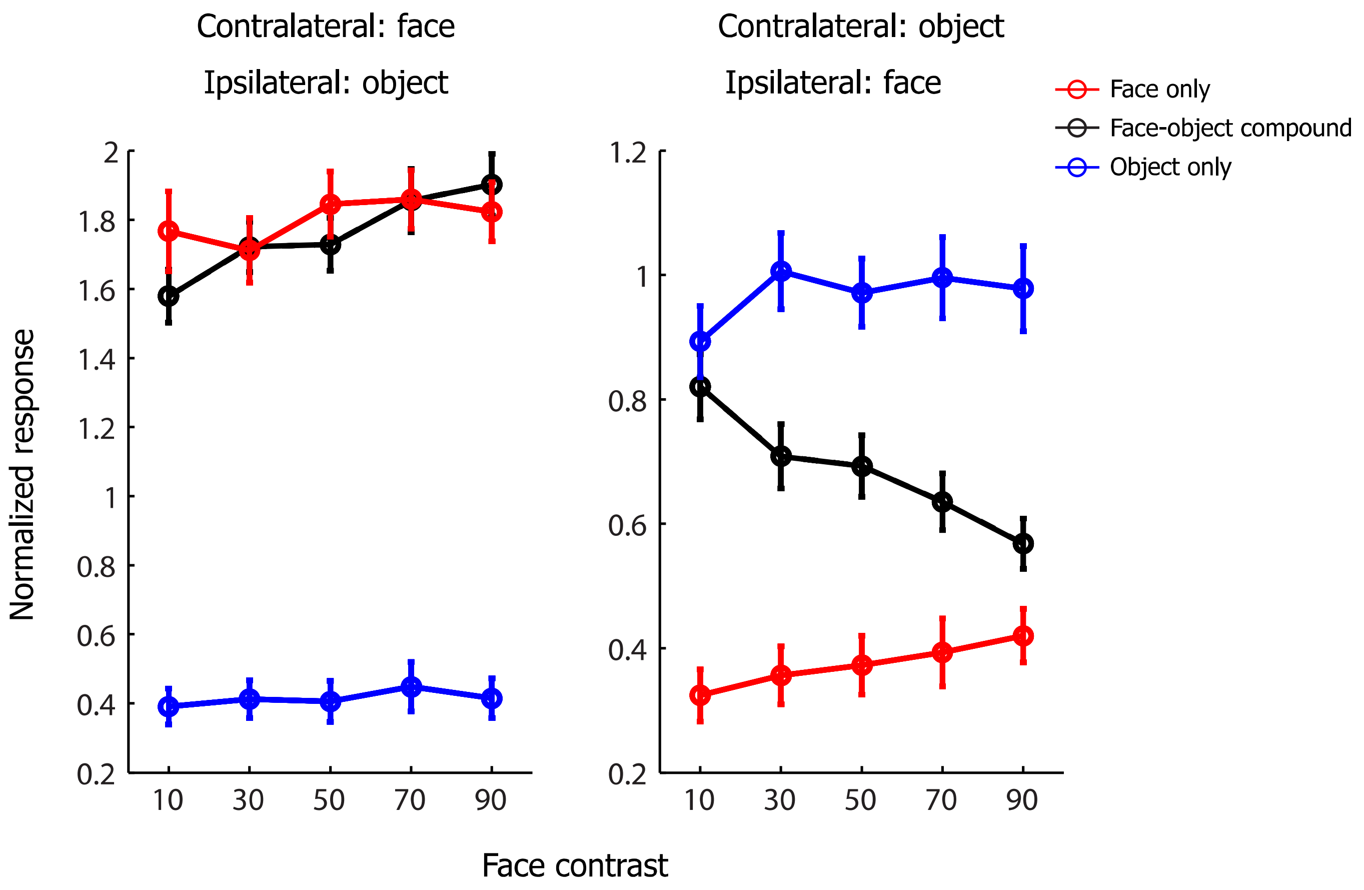

Contralateral: face
Ipsilateral: object
Contralateral: object
Ipsilateral: face
Face only
Face-object compound
Object only
Normalized response
2
1.8
1.6
1.4
1.2
1
0.8
0.6
0.4
0.2
1.2
1
0.8
0.6
0.4
0.2
10
30
50
70
90
10
30
50
70
90
Face contrast

Supplementary Figure 8. Integration rule for subset of face cells showing a greater response to contralateral objects compared to ipsilateral faces. Black line represents response to face-object pair at different relative contrasts. Red (blue) line represents responses to the constituent face (object) of the corresponding face-object pair when presented in isolation.

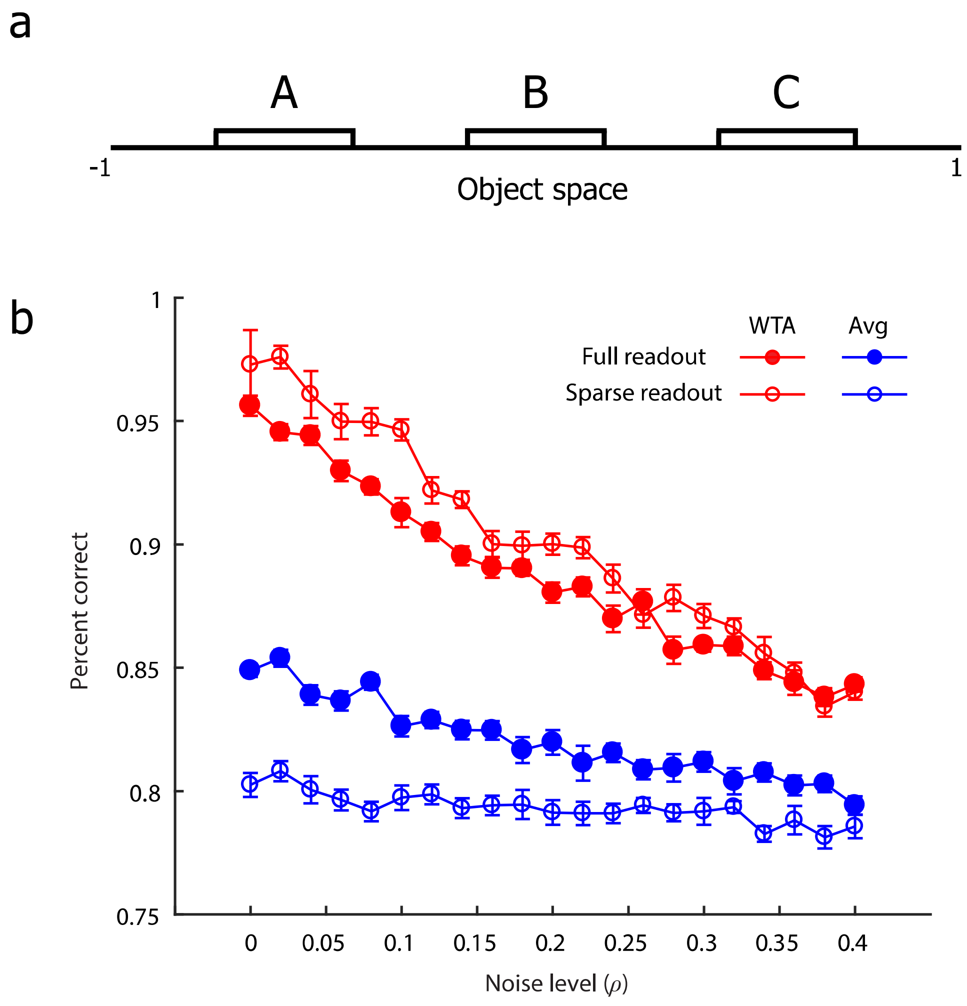

a
A
B
C
-1
1
Object space
b
1
0.95
0.9
0.85
0.8
0.75
Percent correct
WTA
Avg
Full readout
Sparse readout
0
0.05
0.1
0.15
0.2
0.25
0.3
0.35
0.4
Noise level (ρ)

Supplementary Figure 9. Simulation of object decoding as a function of integration rule (winner-take-all, averaging), readout strategy (sparse, full), and noise level. (**a**) Hypothetical one dimensional object space. (**b**) Decoding accuracy (averaged across three classifiers, for A/not A, B/not B C/not C) as a function of noise level, for a winner-take-all rule (red curves) and averaging rule (blue curves). Filled circles indicate performance when reading out all neurons, while open circles indicate performance when reading out neurons selective for the object being detected. The winner-take-all rule always outperformed the averaging rule, with especially pronounced difference for sparse readout.

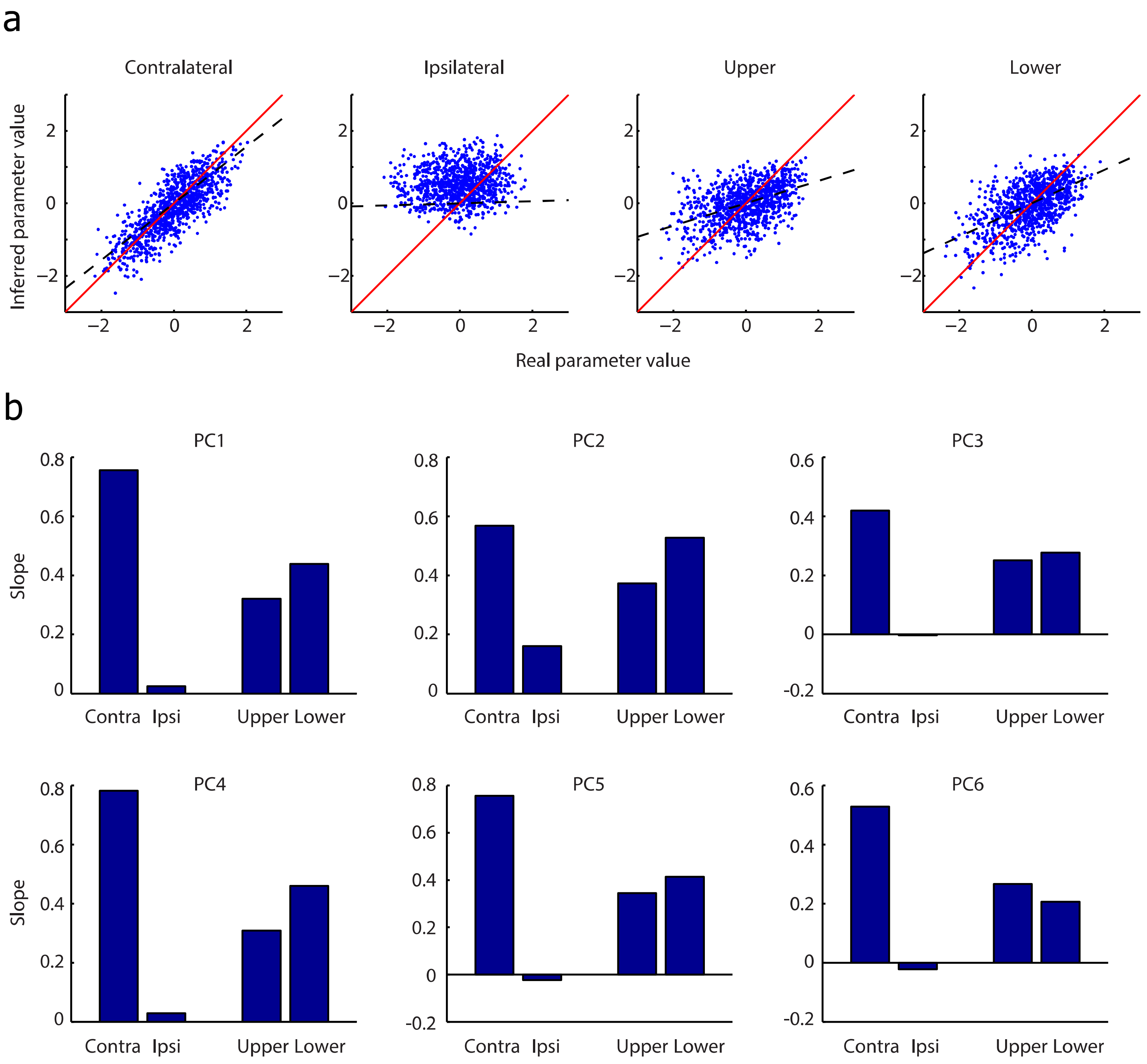
a
Contralateral
Ipsilateral
Upper
Lower
Inferred parameter value
Real parameter value
b
PC1
PC2
PC3
PC4
PC5
PC6
Slope
Contra
Ipsi
Upper
Lower

Supplementary Figure 10. Decoding face features in pairs of faces across different spatial configurations. (**a**) For PC1 (i.e., dimension 1 of our 6-d face space), we plot the predicted versus actual feature value, for a face presented contralaterally or ipsilaterally in a horizontal pair, and for a face presented above or below in a vertical pair. The predicted values were determined according to the decoding model described in the method session: a model of face decoding for pairs of faces. (**b**) The slope of the best-fit line between predicted and actual feature values for each of the 6 dimensions in our 6-d face space, for all four spatial conditions.
